# Enhancing post-operative hypothyroidism treatment: rat thyroid autotransplantation into a pre-vascularized, retrievable cell pouch™ device

**DOI:** 10.3389/fendo.2025.1642916

**Published:** 2025-09-17

**Authors:** Arash Memarnejadian, Pardis Pakshir, Madison Tomlinson, Farideh Berjisian, Ligia Dortas Maffei, Martha Winhall, Ben Muirhead, Jonathan Mofford, Heather Sheardown, Babak Ataei Mehr, Vijay Parikh, Frank R. Shannon, Pericles Calias, Philip M. Toleikis

**Affiliations:** 1 R&D Department, Sernova Biotherapeutics Inc., London, ON, Canada; 2 Department of Chemical Engineering, McMaster University, Hamilton, ON, Canada

**Keywords:** thyroid, transplantation, post-surgery hypothyroidism, triiodothyronine, tetraiodothyronine, thyroid stimulating hormone

## Abstract

**Introduction:**

To overcome the limitations of lifelong hormone replacement therapy for post-operative hypothyroidism, we propose autologous thyroid transplantation into the Cell Pouch™ (CP), an implantable and retrievable medical device that supports vascularization and a homeostatic environment for cell grafting. The CP is currently in clinical trials for islet transplantation in type-1 diabetes, demonstrating its capacity to support long-term graft viability. Here, we apply the CP for thyroid tissue transplantation, leveraging the natural gland’s regulatory capabilities to benefit patients unresponsive to hormone therapy. Building on previous research validating CP’s support for human thyroid grafts in a mouse model, this study evaluates its therapeutic potential in a rat model mimicking clinical thyroidectomy and autologous transplantation.

**Methods:**

We used 24 Lewis rats to assess the CP’s safety and efficacy. After a five-week post-implantation vascularization period, thyroidectomy was performed in 15 rats, and their glands were transplanted into the CP. A non-transplant group (n=4) underwent thyroidectomy only, and a control group (n=5) received no interventions. Hormone levels (total T3, free T4, TSH) were monitored weekly for 22 weeks. Histology and scintigraphy at endpoint evaluated graft function.

**Results and Conclusion:**

Rats with thyroid grafts in the CP restored fT4 and T3 to baseline by weeks 4 and 7, respectively. Explantation reversed this effect. Histological analysis showed well-differentiated follicles with minimal inflammation. Scintigraphy confirmed graft viability. No adverse effects were observed in hematological, liver, or kidney parameters. These findings demonstrate that CP-enabled thyroid transplantation restores function post-thyroidectomy and is safely retrievable, with no residual thyroid tissue, marking a significant safety advancement.

## Introduction

Hypothyroidism, characterized by insufficient production of thyroid hormones, is a common outcome following total and near-total thyroidectomies. These surgeries are typically performed to address conditions such as thyroid cancer, goiter, or refractory hyperthyroidism (1). The current standard treatment for post-surgery hypothyroidism involves lifelong daily administration of exogenous thyroxine (levothyroxine; L-T4), the primary thyroid hormone. This therapy requires constant dose adjustments based on blood thyroxine levels. While effective in normalizing thyroid hormone serum levels, this approach does not replicate the natural thyroid gland’s intricate regulatory mechanisms (2). Long-term L-T4 therapy has been linked to a spectrum of adverse effects, including impaired arterial elasticity and increased left ventricular mass (3), alongside notable changes in lipid profile (4). Beyond these physiological complications, patients often face significant challenges related to frequent hormone dosage adjustments and non-compliance. Furthermore, suboptimal symptom control and a range of side effects, ranging from weight gain and subclinical depression to memory impairment (5), reduce patients’ quality of life and underscore the need for alternative therapeutic strategies (6).

Autologous (auto) transplantation of the healthy portions of the excised thyroid gland has been explored as a potential solution for restoring physiological thyroid function. This concept has been under consideration for over a century (7, 8), with encouraging data from both animal studies (2, 9–11) and small human trials (12–19). However, clinical implementation remains unrealized. Substantial heterogeneity in the methodologies, such as the anatomical location of transplantation, the use of fresh versus cryopreserved tissue grafts, and transplant volume, contribute to this limited utilization. A standardized protocol is needed to address these issues.

In response to these challenges, our research investigates a novel approach using a pre-vascularized Cell Pouch device for thyroid tissue transplantation. The Cell Pouch is a subcutaneously implantable, retrievable, and scalable medical device designed to provide a well-vascularized tissue environment for the transplant, thereby promoting graft survival and function (20, 21). Currently in phase I/II clinical trials for pancreatic islet transplantation in patients with type 1 diabetes, the Cell Pouch has shown promising interim results (Clinical Trial IDs NCT01652911 and NCT03513939).

Our team previously demonstrated the effectiveness of Cell Pouch in maintaining human thyroid tissue viability and functionality over a three-month period using a euthyroid immunocompromised mouse model (22). Building on this foundation, the current proof-of-concept study aims to ascertain the potential applicability of the Cell Pouch system for thyroid autotransplantation. In a rat model, we replicated the process of thyroidectomy and autologous transplantation of excised thyroid tissue into the Cell Pouch device. This design allows the evaluation of the graft’s long-term viability and functional capacity, simulating the clinical scenario within a translational framework.

## Materials and methods

### Animals and study design

This study was approved by the Animal Research Ethics Board, McMaster University (AUP # 22-05-18), in accordance with the national guidelines for the Care and Use of Laboratory Animals.

Twenty-four (24) inbred male albino rats (Lewis strain), with an average age of 6 weeks and a mean weight of 200 ± 20 g, were purchased from Charles River, Canada. Animals were housed in environmentally controlled conditions and fed standardized food and water ad libitum. Rats were assigned to three groups, with details shown in **Table 1**.

**Table 1:** Animals’ group assignment and specification.

Animal Groups	Number of rats	Cell Pouch™ Implant	Thyroidectomy	Transplant
Group 1 (Thyrox+Tx)*	15	Yes	Yes	Yes
Group 2 (Thyrox)	4	Yes	Yes	No
Group 3 (control)	5	Yes	No	No

*Thyrox, thyroidectomized; Tx, transplanted with autologous thyroid tissue.

**Figure 1:**
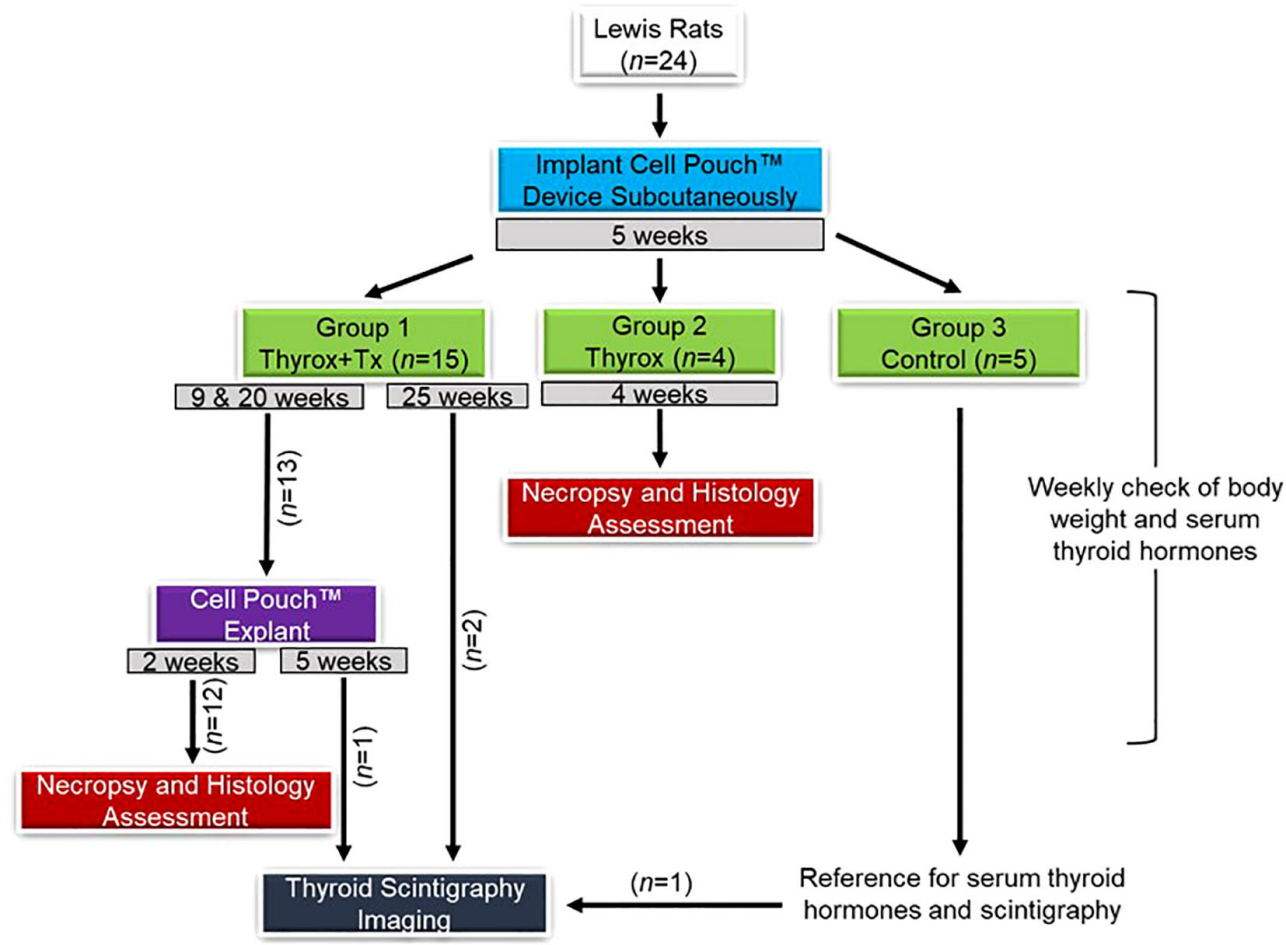
Overview of the experimental design. Lewis rats (n=24) were assigned to three groups: Group 1 (Thyrox+Tx, n=15) underwent thyroidectomy followed by autotransplantation into a subcutaneously implanted Cell Pouch; Group 2 (Thyrox, n=4) had thyroidectomy without transplantation; Group 3 served as the untreated control (n=5). Thyroid hormone levels were assessed weekly. The Cell Pouch was explanted at specified intervals for necropsy and histology, with thyroid scintigraphy performed at week 25.

All rats were implanted with the Cell Pouch device. After 5 weeks, the thyroid glands of animals in groups 1 (Thyrox+Tx) and 2 (Thyrox) were surgically removed. The excised glands were immediately autotransplanted into the vascularized Cell Pouch device in group 1 rats. Group 2 rats did not receive the transplants. Animals in group 3 (Control) were sex- and age-matched, had neither the thyroidectomy nor the transplantation and served as controls for blood hormone levels. As shown in **Figure 1**, all animals were weekly monitored for their level of thyroid hormones. The Cell Pouch device was explanted from the Thyrox+Tx animals at 9 (n=3) and 20 weeks (n=10) post-transplant. Explanted animals were kept alive for two additional weeks, and their level of blood hormones was monitored. Three representative animals from the Thyrox+Tx group and one control rat were imaged for radioisotope uptake at the 25-week time point. Explanted Cell Pouch devices were histologically examined.

### Surgical procedures

#### Cell pouch™ implantation

One Single-Channel Mini-Cell Pouch™ device was surgically inserted into the subcutaneous space of the abdominal region in rats five weeks before the total thyroidectomy/transplantation procedure. The pre-implantation period allowed the formation of a vascularized tissue chamber within the device scaffold. The surgical procedure was carried out on isoflurane-anesthetized animals under sterile conditions, involving placing the device into a pre-established subcutaneous pocket, followed by fixation to the underlying rectus abdominus muscle. A representative image of a rat with the Cell Pouch implanted subcutaneously in the abdominal region is shown in **Supplementary Figure 1**.

#### Total thyroidectomy and transplantation

The rats were anesthetized with an intraperitoneal injection of ketamine hydrochloride (75 mg/kg) and xylazine hydrochloride (6 mg/kg). For thyroidectomy, an 8 cm longitudinal incision into the skin and platysma muscle was made along the cranial-caudal midline of the ventral cervical region. Using a binocular microscope, a blunt dissection was performed to separate the sternocleidomastoid and sternohyoid muscles, exposing the trachea. The pair of parathyroid glands was identified on the cranial lateral pole of each thyroid gland lobe, dissected away from the thyroid gland and implanted into the pocket made in the sternohyoid musculature. The isthmus of the thyroid gland was elevated and incised, while both lobes of the thyroid gland were excised, preserving the laryngeal nerves and vasculature. Thyroid tissues were placed in sterile saline within a petri dish, and the surgical wound was closed with metal staples. For transplantation, the Cell Pouch™ opening zone was accessed through a 1 cm transverse incision in the abdominal skin. The Cell Pouch™ plug was removed, and both excised thyroid lobes, cut into 2mm pieces, were inserted into the empty chamber of the device. The opening of the Cell Pouch™ was then sutured closed, as well as the skin incision. Animals were given post-operative analgesics (buprenorphine, 0.02 mg/kg and carprofen, 5 mg/kg, both subcutaneous) and recovered.

### Thyroid hormones, hematology, and biochemistry measurements

In accordance with the approved Animal Use Protocol, we collected 0.7 mL of blood from the tail artery every week for 22 weeks, starting one week prior to total thyroidectomy and transplantation. Isolated sera were stored at -80°C until they were used for the in-house measurement of thyroid-stimulating hormone (TSH), free thyroxine (fT4) and total triiodothyronine (T3) levels. Rat TSH ELISA kit (ALPCO, Cat # 55-TSHRT-E01) with an analytical measuring range of 0.1 to 80 ng/mL, rat fT4 competitive ELISA kit (Antibodies-Online, Cat # ABIN6966896) with an analytical measuring range of 1.563 to 100 pg/mL and rat T3 competitive ELISA kit (Antibodies-Online, Cat # ABIN6970859) with an analytical measuring range of 0.156 to 10 ng/mL were used.

Serum samples were also shipped to Antech^®^ Diagnostics (Ontario, Canada) to determine the total calcium level (mm/L) one week before (baseline) and one week after the thyroidectomy to ensure that reimplanted parathyroid glands were functional. Complete blood cell count (CBC), liver (bilirubin, alkaline phosphatase, and alanine transaminase) and kidney (blood urea nitrogen and creatinine) function tests were also performed by Antech^®^ Diagnostics at baseline and week 20 post-transplantation for procedure safety assessment.

### Scintigraphy imaging

Imaging was performed for representative rats 25 weeks post-thyroidectomy and transplantation in the STTARR Facility, University Health Network, Toronto. Rats were injected intravenously with 0.5 mCi of ^99m^Tc-Pertechnetate and imaged using a Mediso^®^ Nanoscan SPECT/PET/CT instrument 20–35 mins post-injection. Animals were euthanized after the imaging procedure. The mean percentage of injected dose per gram (%ID/gram) was calculated as the region of interest (ROI) activity divided by the injected dose multiplied by 100.

### Histopathology

At 20 weeks post-transplantation, the Cell Pouch™ devices were explanted from the animals in survival surgeries, fixed in 10% neutral buffered formalin, dehydrated and embedded in paraffin blocks. Five-micron-thick sections were stained with hematoxylin and eosin (H&E). Sequential sections were used for immunofluorescence and immunohistochemical staining to detect the following markers:

TG (anti-thyroglobulin antibody, AbCam, Cat # ab156008, 1/100 dilution), thyroid peroxidase (TPO, anti-thyroid peroxidase antibody, AbCam, Cat #ab278525, 1/100 dilution), von-Willebrand Factor (vWF, anti-WF, DAKO Agilent, Cat #A008202-2, 1/1400 dilution), and Ki67 (anti-Ki67 antibody, AbCam, Cat #ab16667, 1/50 dilution). The Click-iT™ Plus TUNEL Assay Kit for *In Situ* Apoptosis Detection (Thermo Fisher Scientific, Cat # C10619) was used per the manufacturer’s instructions with recommended positive and negative controls.

### Statistical analysis

The average fold change of the thyroid hormone levels was compared to the animal’s pre-transplant (baseline) levels. A Mann-Whitney non-parametric U-test was used to compare hormone levels between the control and transplanted animals at the 20-week post-transplant timepoint. The non-parametric Wilcoxon Signed-Rank test was used to compare serum hematological and biochemical values between the baseline and 20 weeks post-transplantation. *p<0.05* was considered statistically significant.

## Results

### Kinetics of thyroid hormones in thyroidectomized and transplanted animals

**Figure 2:**
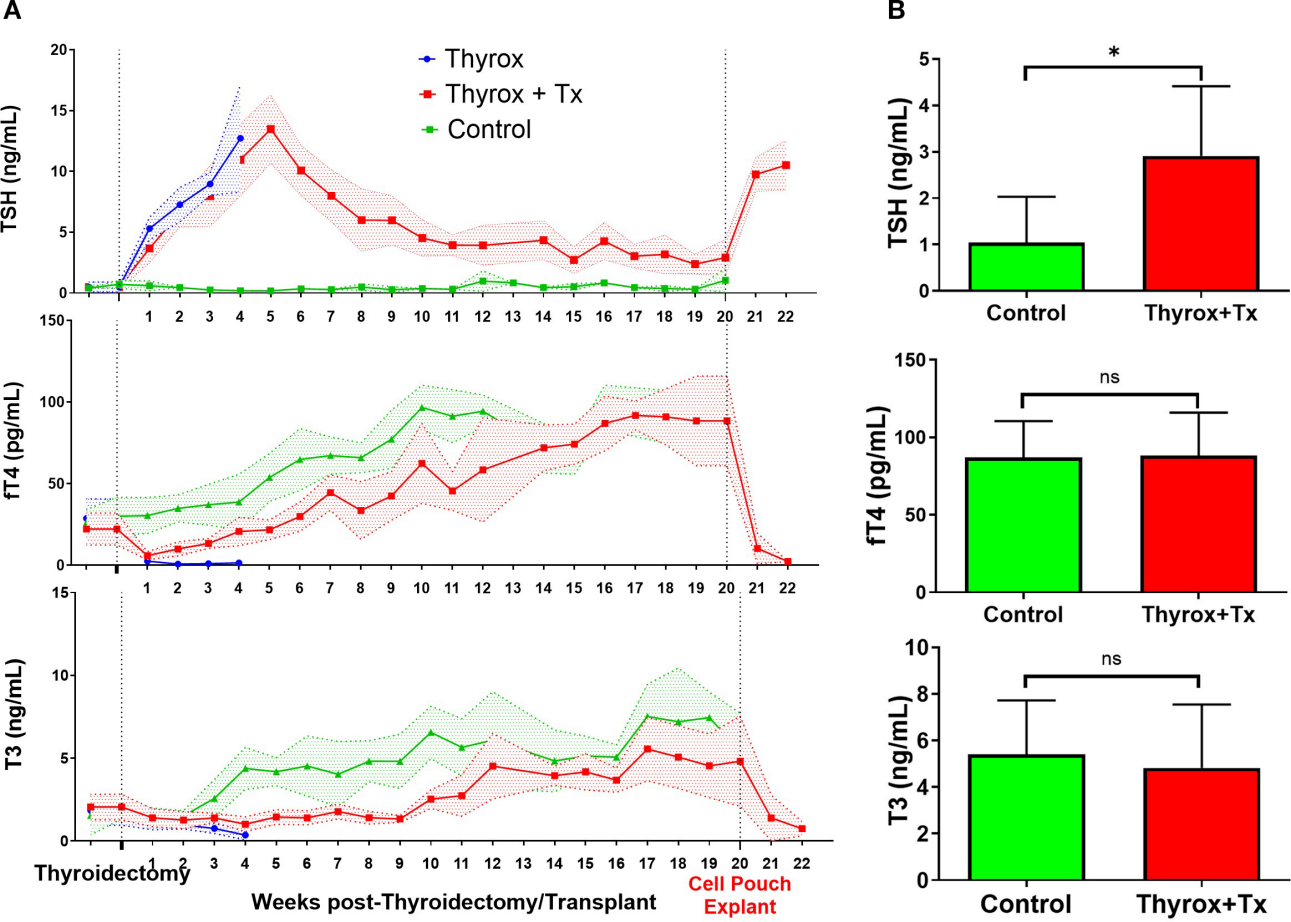
Thyroid hormone profiles Over Time and at Endpoint. **(A)** Hormone levels (TSH, fT4, and T3) are presented for Thyrox (n=4), Thyrox+Tx (n=15), and Control (n=5) groups. The profiles extend from baseline up to 20 weeks post-thyroidectomy and include two additional weeks following Cell Pouch explant. In the Thyrox+Tx group, data from weeks 9 to 22 are derived from the remaining 12 subjects due to the early explantation of 3 animals. **(B)** Displays a week 20 hormone level comparison between Control (n=5) and Thyrox+Tx (n=12) groups. Data in both panels reflect mean ± SD. A star indicates *p*<0.05 significance, and “ns” marks non-significance.

Serum thyroid hormone levels were assessed at two initial time points (baseline), *i.e*., one week and one hour before thyroidectomy (Thyrox) and/or transplantation (Tx) and subsequently monitored weekly throughout the study. Measurements continued post-explantation of the Cell Pouch device in a survival surgery, with rats undergoing further hormonal evaluation for an additional two weeks (**Figure 1**). As shown in **Figure 2A**, all animals in groups one (Thyrox+Tx) and two (Thyrox) had an immediate increase in TSH and a decline in T3/fT4 levels following the thyroidectomy operation. In the Thyrox group that did not receive the thyroid transplant, hormone levels did not return to baseline within four weeks, at which point they were euthanized due to significant weight loss (weight data not included). The average fold change in hormone levels compared to baseline in the Thyrox group was 26 for TSH, 0.05 for fT4, and 0.2 for T3, measured four weeks after thyroidectomy. Conversely, rats that received autotransplantation of their excised thyroid tissue into the Cell Pouch (Thyrox+Tx group) showed a restoration of fT4 and T3 levels to baseline at approximately 4 and 7 weeks post-transplantation, respectively. Throughout the 20-week study period, the levels of fT4 and T3 in the Thyrox+Tx group remained comparable to those in the age and sex-matched naïve control rats (Control group). At the 20-week mark, a comparison of fT4 and T3 levels between the Thyrox+Tx and Control groups, as illustrated in **Figure 2B**, revealed no considerable differences. In the Thyrox+Tx group, TSH levels peaked up to 28-fold at week 5, subsequently decreasing to a stable level around week 11. By week 20 post-transplant, the average TSH level in the Thyrox+Tx group was approximately five times the baseline and significantly higher than the TSH in the control group (2.91 ± 1.5 vs 1.04 ± 0.9, p=0.047). Following the removal of the device, animals quickly reverted to hypothyroidism, characterized by increased TSH and decreased fT4/T3 levels. This pattern indicates that the hormones detected in the bloodstream were primarily sourced from the Cell Pouch-thyroid graft.

### Histopathological evaluation of thyroid transplants

**Figure 3:**
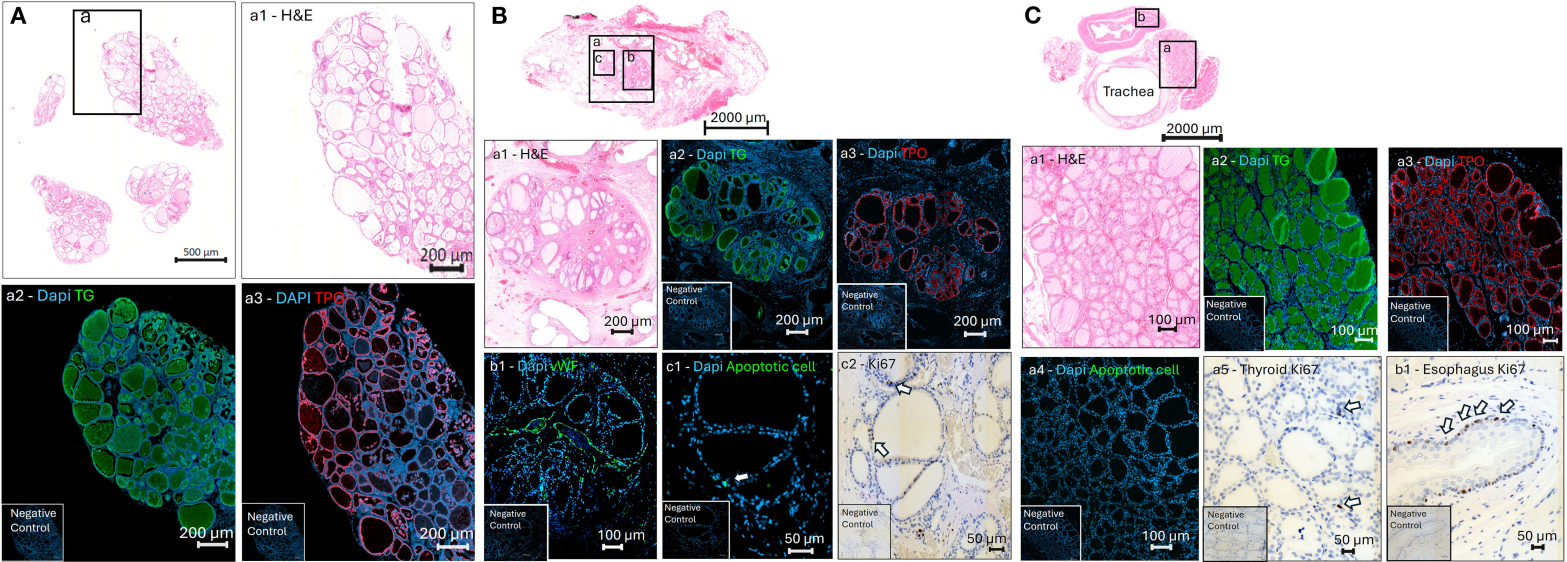
Histological and immunohistochemical analysis of native and transplanted thyroid tissue. **(A)** Native rat thyroid dissected into small pieces: H&E staining shows preserved follicular architecture (a1); immunofluorescence confirms thyroglobulin (TG, green, a2) and thyroid peroxidase (TPO, red, a3) expression within follicles. **(B)** Cross-section of Cell Pouch 20 weeks post-transplantation: H&E reveals intact thyroid follicles (a1); immunofluorescence confirms TG (a2) and TPO (a3) expression; vWF staining indicates vascularization (b1); low apoptosis detected by apoptotic marker (green, c1, white arrow); sparse Ki67-positive proliferating cells are visible (c2, white arrows). **(C)** Naïve rat neck section showing thyroid **(a)**, esophagus **(b)**, and trachea. Thyroid region displays healthy follicles by H&E (a1), TG (a2), and TPO (a3) staining; minimal apoptosis (a4) and rare Ki67+ cells (a5) indicate low turnover. Esophageal epithelium (b1) is a positive control for proliferation with abundant Ki67+ nuclei (white arrows). DAPI (blue) marks nuclei across all panels. Negative controls are shown in the insets.

At weeks 9 and 20 post-transplantation, Cell Pouch devices retrieved from the Thyrox+Tx rats, along with thyroid glands harvested with the trachea of naïve control rats, were histologically assessed and compared (**Figure 3**). Histological examination of hematoxylin and eosin (H&E)-stained sections revealed that the internal architecture of the Cell Pouch chambers consisted of a loosely organized fibroblastic stroma, punctuated by a network of neo-vessel formation, including small capillaries and larger vascular structures. Instances of dense collagenous stroma were observed to be mild and infrequent, with no discernible differences between control and transplanted specimens. Examination of the Cell Pouch sections disclosed that the transplanted thyroid tissue maintained well-differentiated follicular structures, characterized by microfollicular and macrofollicular formations, with relatively normal colloid densities and mild follicular cell hyperplasia. Inflammation was minimal, and there were no indications of autoimmune thyroiditis, findings that were consistent across both control and transplanted groups. Immunofluorescence (IF) staining of the Cell Pouch grafts highlighted the presence of thyroid-specific markers. Notably, thyroid peroxidase (TPO) and thyroglobulin (TG) were detected within viable thyroid follicles in dissected tissue before transplantation, after transplantation to the Cell Pouch and in the intact thyroid glands of control rats (**Figures 3A–C**). Additionally, endothelial cell marker von Willebrand Factor (vWF) staining confirmed extensive vascularization within the tissues of the Cell Pouch structure (**Figures 3B-b1**). A TUNEL assay, employed to assess apoptotic cell patterns, showed normal, non-apoptotic profiles in Cell Pouch grafts, analogous to those in the intact thyroid glands from control rats (**Figures 3B-c1 C-a4**). Further, immunohistochemical (IHC) staining for Ki67, a marker of cellular proliferation, indicated similar proliferative activity within both Cell Pouch transplants and the intact thyroid glands of control subjects (**Figures 3B-c2, C-a5**). Ki67 staining efficacy was validated using a control rat esophagus section (**Figures 3C-b1**).

### Scintigraphy findings

**Figure 4:**
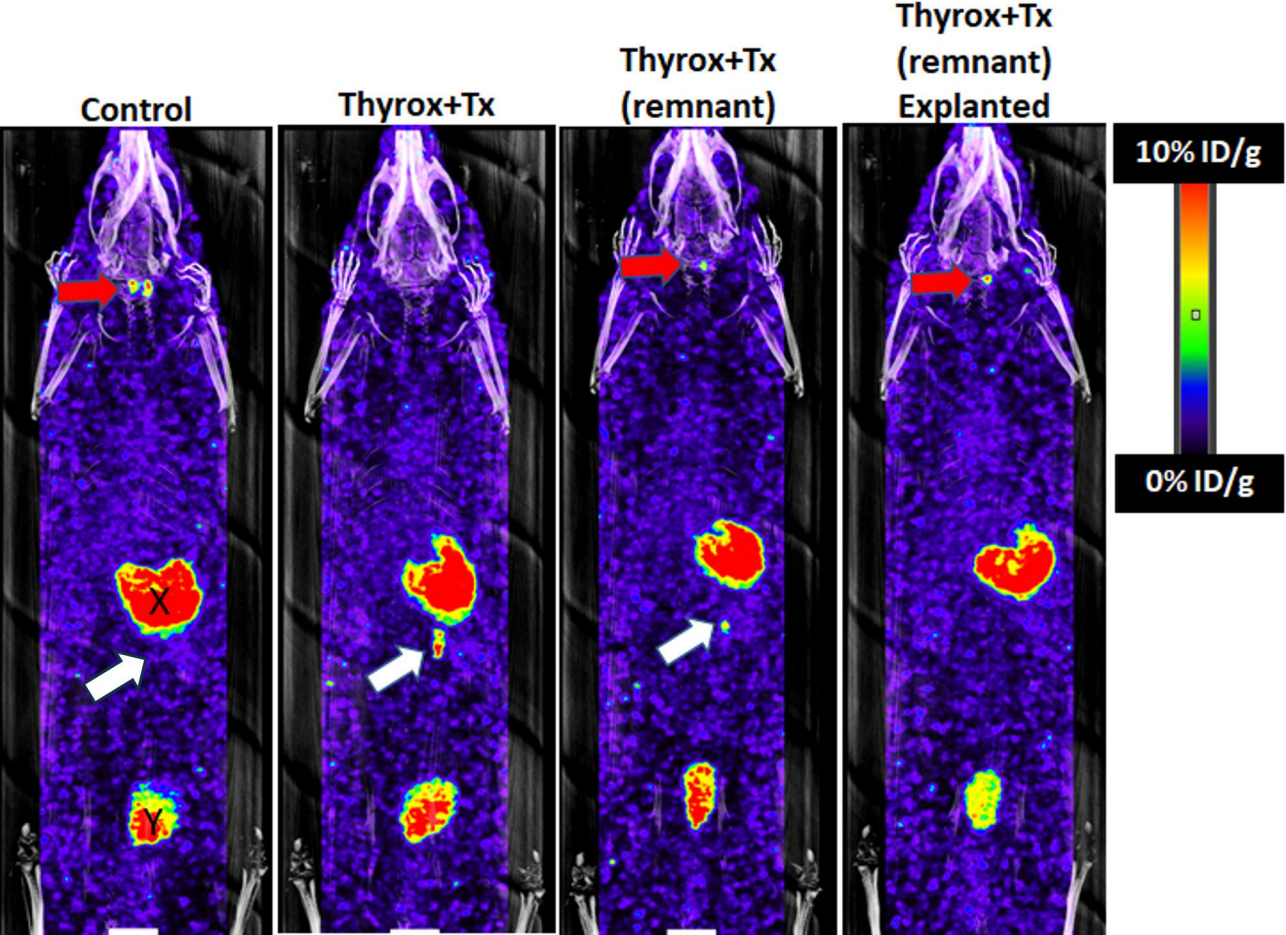
Whole-body scintigraphy comparing isotope uptake. The control shows typical thyroid uptake (red arrow) and no uptake in the Cell Pouch (device only, white arrow) implant site. In Thyrox+Tx subjects, reduced or absent uptake in the thyroid bed indicates total thyroidectomy or residual lobe function, respectively. Significant uptake in the Cell Pouch area (white arrows) is evident, which ceases after the pouch’s removal (explant), signifying the graft’s confinement to the device. “X” and “Y” mark the epigastric and bladder, known for high technetium uptake, respectively.

Scintigraphy assessments were conducted 25 weeks after thyroidectomy and transplantation procedures on a subset of four randomly selected rats (refer to **Figure 1**). This subset comprised three rats from the experimental group (Thyrox+Tx) and one from the control group. According to the data presented in **Figure 4**, the control group rat demonstrated effective radiotracer uptake in both thyroid lobes and no uptake in the Cell Pouch device implantation site. Conversely, the first rat in the Thyrox+Tx group displayed no radiotracer uptake in the neck region but exhibited significant uptake within the transplanted graft, indicating graft functionality. The second experimental rat showed residual thyroid activity in the left lobe of the neck and significant graft uptake. The third rat, selected from those having undergone Cell Pouch removal via survival surgery at 20 weeks post-transplantation, underwent scintigraphy imaging five weeks after the Cell Pouch was explanted. This animal exhibited no uptake at the graft site, validating the surgical confinement of the transplant to the removed Cell Pouch. This animal also retained residual thyroid tissue in the left lobe. **Table 2** summarizes the mean uptake of ^99m^TcO_4_ in both the Cell Pouch and neck regions for these subjects. Analysis of the uptake ratio between the Cell Pouch and glandular tissue in rats with residual thyroid tissue revealed substantial functionality of the Cell Pouch, evidenced by significant radiotracer absorption.

**Table 2:** ^99m^TcO_4_ uptake levels and the ratio between the Cell Pouch and thyroid gland in representative animals.

Animal Groups	Mean ^99m^TcO_4_ Uptake (%ID/gram)*		Uptake Ratios
	Cell Pouch	Thyroid Glands	Cell Pouch to Thyroid Glands
Control	0.32	4.39	0.07
Thyrox+Tx	4.89	0.22	22.22
Thyrox+Tx (remnant)	3.95	3.26	1.21
Thyrox+Tx (remnant) Explanted	0.22	4.37	0.05

*%ID/gram: the mean percentage of injected dose per gram.

### Hematological and biochemical safety analysis

To assess the safety profile of our proposed strategy, we performed a comprehensive evaluation on all subjects in group 1 (Thyrox+Tx, n=15). This included a detailed analysis of hematological and serum biochemical parameters, such as complete blood cell counts, hemoglobin levels, and liver and kidney function tests. Baseline measurements were established one week before the initiation of Thyrox+Tx, with follow-up assessments conducted at the conclusion of the study, 20 weeks post-treatment. Serum calcium levels were checked at baseline and one week post-thyroidectomy as an indicator of the function of reimplanted parathyroid glands. Our analysis revealed no statistically significant changes in the hematological parameters, calcium levels, and liver function tests (including bilirubin, alkaline phosphatase, and alanine transaminase) from baseline to study end. However, we observed a notable but non-pathological increase in the serum creatinine and blood urea nitrogen (BUN) levels, although these values remained within normal limits (**Supplementary Figure 2**). The underlying cause of these elevated levels might be an age-related decline in kidney function, the effects of weekly repeated blood draws or stress on the animals.

## Discussion

People treated for endocrine deficiencies, including hypothyroidism, hypoparathyroidism, and diabetes mellitus, currently rely on daily replacement therapies. These treatments, however, are inherently limited due to the absence of a natural autoregulatory system, often leading to suboptimal outcomes and discord between the therapy and the patient’s real-time physiological needs. Moreover, factors such as complex dosing schedules, side effects, and the challenges of daily life integration can diminish patient compliance, undermining the therapy’s effectiveness. This highlights the urgent need for innovative cell therapy approaches (23).

Daily L-T4 therapy is a widely available and low-cost standard of care for patients with hypothyroidism (including post-thyroidectomy). Despite this, up to 10% of these patients report persistent clinical symptoms (24, 25), suggesting inadequacy of the current treatment standard. The complexity of this issue is compounded by the fact that many symptoms, such as memory impairment, depression, and weight gain, are nonspecific and can often be attributed to nonthyroidal causes, resulting in an underestimation of the patient’s condition when they are within the biochemical target range of TSH. The reliance on L-T4 therapy as the sole treatment option has become a deeply ingrained clinical dogma, primarily due to its affordability and the lack of therapeutic alternatives (26). This one-size-fits-all approach overlooks the complexity of thyroid hormone dynamics and the individual variability in hormone metabolism and sensitivity (6, 26). The persistent symptoms experienced by a considerable subset of patients under L-T4 therapy underscore the necessity for alternative therapeutic strategies that can more closely mimic the natural physiology of thyroid hormone regulation. Efforts to enhance patient outcomes and more closely replicate the body’s natural thyroid function have led to the proposition of combining T3 and T4 therapies. Despite the potential benefits that T3 and T4 combination therapy may offer over T4 monotherapy for some hypothyroid patients, particularly those with specific metabolic needs or genetic backgrounds (27), more than a dozen clinical trials have failed to prove its superior benefits (28). While evidence suggests certain advantages of combination therapy, there is conflicting data indicating no substantial improvement in patients’ well-being, cognitive function, or quality of life (29). Additionally, combination therapy can lead to adverse effects, including anxiety, nausea, and compromised bone health. The decision to opt for this therapy should be highly personalized, taking into account patient-specific factors such as genetic markers and individual responses to prior treatments (29).

The growing voice of patient advocacy groups, found through simple internet searches, highlights the widespread recognition and concern over this issue within the hypothyroid community. These groups advocate for greater attention to the qualitative aspects of hypothyroidism treatment, emphasizing that the current metrics of success, primarily based on normalizing TSH hormone levels, may not adequately capture the full spectrum of patient well-being and quality of life. Adding to the complexity, some patients who continue to experience hypothyroid symptoms while on L-T4 report symptom improvement when switched to desiccated thyroid extract (DTE), a traditional treatment derived from pig thyroid glands (30), after not responding adequately to L-T4 therapy. However, DTE comes with its own set of challenges, including concerns over its regulation and the lack of comprehensive safety and efficacy data (31). The concept of replacing the thyroid gland, disregarding whether it is removed, ablated, or dysfunctional, is recognized as a viable option but has not become a routine clinical practice. Previous research highlights a wide range of inconsistencies in critical factors, including the pathology of thyroid transplants, using fresh *versus* frozen tissue, the volume of tissue, the preferred transplantation site, and overall procedural specificities (8). Our innovative approach integrates the vascularized, retrievable Cell Pouch device, offering multiple clinical benefits. The unique ability to promote vascularization ensures a robust blood supply, which supports graft viability and function (22). When choosing a transplantation site, various ectopic locations, such as the forearm, thigh, and neck muscles, have been explored. Each site presents unique challenges: the forearm, while easily accessible for monitoring, may increase scar visibility and patient discomfort; the thigh, though more concealed, complicates monitoring and might interfere with daily activities. Transplanting thyroid tissue to neck muscles also brings specific concerns, notably the proximity to vital areas such as airways and critical vasculature, which could pose risks should hyperplasia of the transplanted tissue occur. Designed for implantation in the abdominal area under the deep fascia, the Cell Pouch device strategically minimizes visibility and physical discomfort while reducing the risk of displacement. Its subcutaneous positioning allows for straightforward monitoring, access and retrieval, which are crucial in cases of complications, suboptimal outcomes, or postoperative recurrent hyperthyroidism. Scintigraphy data demonstrate no local radioisotope uptake after the removal of the Cell Pouch (**Figure 4**), confirming that the thyroid transplant remains entirely confined within the device. This is particularly important in cases of thyroid autotransplantation, where there is a risk of transplanting non-visible malignant cells. The possibility of implanting undetected thyroid cancer in muscle is a concern. In a recent study, 23.1% of cervical multinodular goiters that had a benign preoperative fine needle aspiration were found to have thyroid cancer on final pathology (32). This underscores the importance of using a containment system that not only supports graft viability but also permits removal in the event of unexpected pathology. The design of the Cell Pouch mitigates this risk by facilitating prompt and uncomplicated retrieval of the contained transplanted tissue if malignancy is detected. This feature significantly enhances patient safety by ensuring that any potential risks can be addressed swiftly and effectively. These characteristics make the Cell Pouch an advanced solution in thyroid autotransplantation by merging efficacy with enhanced safety features.

In the current study, we aimed to perform total thyroidectomies for a better evaluation of the thyroid autograft without the possible interference of thyroid remnants. However, it was observed that 5 out of the 15 rats that underwent thyroidectomy (approximately 33%) retained thyroid tissue remnants, evidenced by scintigraphy imaging (**Figure 4**) and histological assessment of the trachea (data not shown). Imaging data revealed that over 50% of thyroid functionality could be attributed to the Cell Pouch graft (**Table 2**). Moreover, there was no statistically significant difference in the level of thyroid hormones between the transplanted animals with a total thyroidectomy and those with remnants (data not shown). These data showed that Cell Pouch grafts effectively restored thyroid hormone homeostasis, regardless of the presence of thyroid tissue remnants. The occurrence of residual thyroid tissues in human total thyroidectomies is a well-documented phenomenon (33). Thus, our findings reflect the clinical reality, providing further relevance to our study.

Our results showed that restoration of fT4 and T3 was accompanied by a remarkable decrease in TSH levels, indicating the activation of the thyroid-pituitary axis’s negative feedback mechanism. Nevertheless, in euthyroid animals, TSH levels remained somewhat elevated throughout the 20-week duration of the study, as depicted in **Figure 2**. This finding aligns with previous literature indicating that while thyroid transplantation can sustain euthyroidism, it may concurrently provoke enhanced activity within the anterior pituitary gland (16, 17). Such studies have highlighted a lag in normalizing TSH levels following thyroid transplantation. While our study was sufficiently long to observe animals achieving a euthyroid state, the duration was not enough to witness the complete normalization of TSH levels, highlighting a limitation in our study design regarding the assessment timing. Recent progress in understanding thyroid hormone regulation underscores the complexity and multiplicity of factors involved in the feedback interaction between the thyroid and pituitary glands (34). These insights challenge the notion of TSH as a definitive marker of euthyroidism or as a precise indicator for the adjustment of thyroid function in patients treated with L-T4 (35), suggesting that this may also extend to cases of thyroid transplantation.

Advances in stem cell biology and the differentiation of stem cells into endocrine cells, including functional thyrocytes (36), have opened new avenues for developing a functional cure for post-surgical hypothyroidism through cell therapy. Combining the Cell Pouch device with allogeneic stem cell-derived thyrocytes offers a potential alternative for patients with thyroid malignancies where autologous thyroid glands cannot be transplanted. This strategy requires the development of immune-evasive stem cells, which is currently in its early stages. Until this is achieved, thyroid autologous transplantation could be a promising treatment, especially for thyroidectomized patients who may not respond to L-T4 therapy. Additionally, the concept of cryopreserving thyroid tissue for subsequent autologous transplantation presents a viable option for those unresponsive to L-T4 treatment. While our current study did not explore this possibility, it is an area of focus in our ongoing research efforts.

In summary, our study provides compelling evidence that thyroid autotransplantation into the Cell Pouch device following thyroidectomy is effective in restoring thyroid hormone levels. These findings justify further exploration of this concept in clinical trials.

## Data Availability

The original contributions presented in the study are included in the article/**Supplementary Material**. Further inquiries can be directed to the corresponding author.

## Supplementary material

Supplementary Figure 1 Subcutaneous implantation of the Cell Pouch™ in the abdominal region of a rat. The image shows a representative rat after the Single-Channel Mini-Cell Pouch™ device placed in the subcutaneous abdominal space. The rectangular outline indicates the position of the implanted device beneath the skin.

Supplementary Figure 2 Comparative analysis of hematological and serum biochemical parameters. Blood tests were done at the baseline (one week before thyroidectomy and transplantation) and before the animals were euthanized (20 weeks post-thyroidectomy and transplantation). The non-parametric Wilcoxon Signed-Rank test was used to compare the values between the two time points. *P* values are indicated for each graph. Dash lines show the lower and upper normal limits.
