# Correction: Enhancing post-operative hypothyroidism treatment: rat thyroid autotransplantation into a pre-vascularized, retrievable cell pouch™ device

**DOI:** 10.3389/fendo.2025.1743546

**Published:** 2025-12-01

**Authors:** Arash Memarnejadian, Pardis Pakshir, Madison Tomlinson, Farideh Berjisian, Ligia Dortas Maffei, Martha Winhall, Ben Muirhead, Jonathan Mofford, Heather Sheardown, Babak Ataei Mehr, Vijay Parikh, Frank R. Shannon, Pericles Calias, Philip M. Toleikis

**Affiliations:** 1 R&D Department, Sernova Biotherapeutics Inc., London, ON, Canada; 2 Department of Chemical Engineering, McMaster University, Hamilton, ON, Canada

**Keywords:** thyroid, transplantation, post-surgery hypothyroidism, triiodothyronine, tetraiodothyronine, thyroid stimulating hormone

Authors Martha Winhall and Heather Sheardown were omitted as authors in the published article. The correct author list reads:

“Arash Memarnejadian^1,*,^ Pardis Pakshir^1^, Madison Tomlinson^1^, Farideh Berjisian^1^, Ligia Dortas Maffei^1^, Martha Winhall^1^, Ben Muirhead^2^, Jonathan Mofford^2^, Heather Sheardown^2^, Babak Ataei Mehr^1^, Vijay Parikh^1^, Frank R. Shannon^1^, Pericles Calias^1^, Philip M. Toleikis^1^

^1^ R&D Department, Sernova Biotherapeutics Inc., London, ON, Canada

^2^ Department of Chemical Engineering, McMaster University, Hamilton, ON, Canada”

The **Author Contributions** Statement has been corrected to read:

“AM: Investigation, Formal Analysis, Conceptualization, Data curation, Project administration, Writing – review & editing, Methodology, Supervision, Writing – original draft. PP: Methodology, Conceptualization, Project administration, Writing – review & editing. MT: Formal Analysis, Data curation, Methodology, Writing – review & editing. FB: Writing – review & editing, Methodology. LM: Writing – review & editing, Methodology. MW: Investigation, Conceptualization, Writing – review & editing. BM: Methodology, Writing – review & editing. JM: Writing – review & editing, Methodology. HS: Methodology, Writing – review & editing. BAM: Data curation, Writing – review & editing, Methodology. VP: Project administration, Writing – review & editing. FS: Conceptualization, Writing – review & editing. PC: Writing – review & editing. PT: Investigation, Conceptualization, Writing – review & editing, Supervision”.

Authors Martha Winhall and Heather Sheardown were mistakenly included in the **Acknowledgements** section. Both individuals made substantial contributions to the study and should have been listed as co-authors. The **Acknowledgements** section, “The authors would like to thank Drs. Martha Winhall and Heather Sheardown for their dedication to this study,” has therefore been removed in the corrected version.

The conflict of interest statement has been corrected to include author Martha Winhall’s commercial affiliation: “This research was funded by Sernova Biotherapeutics Inc., a publicly traded company. The funder had the following involvement in the study: study design, conduct and data analysis. Authors AM, PP, MT, FB, LM, MW, BA, VP, FS, PC and PT were employed by the company Sernova Biotherapeutics Inc and may hold equity or stock options in the company. Sernova Biotherapeutics Inc. owns the intellectual property related to the Cell Pouch™ medical device and therapeutic cells.”

The original version of this article has been updated.

