# Supplementary figures and images for: Enhancing post-operative hypothyroidism treatment: rat thyroid autotransplantation into a pre-vascularized, retrievable cell pouch™ device
Source: Front Endocrinol (Lausanne). 2025 Sep 17;16:1642916. doi: 10.3389/fendo.2025.1642916 (PMC12483921; doi:10.3389/fendo.2025.1642916)

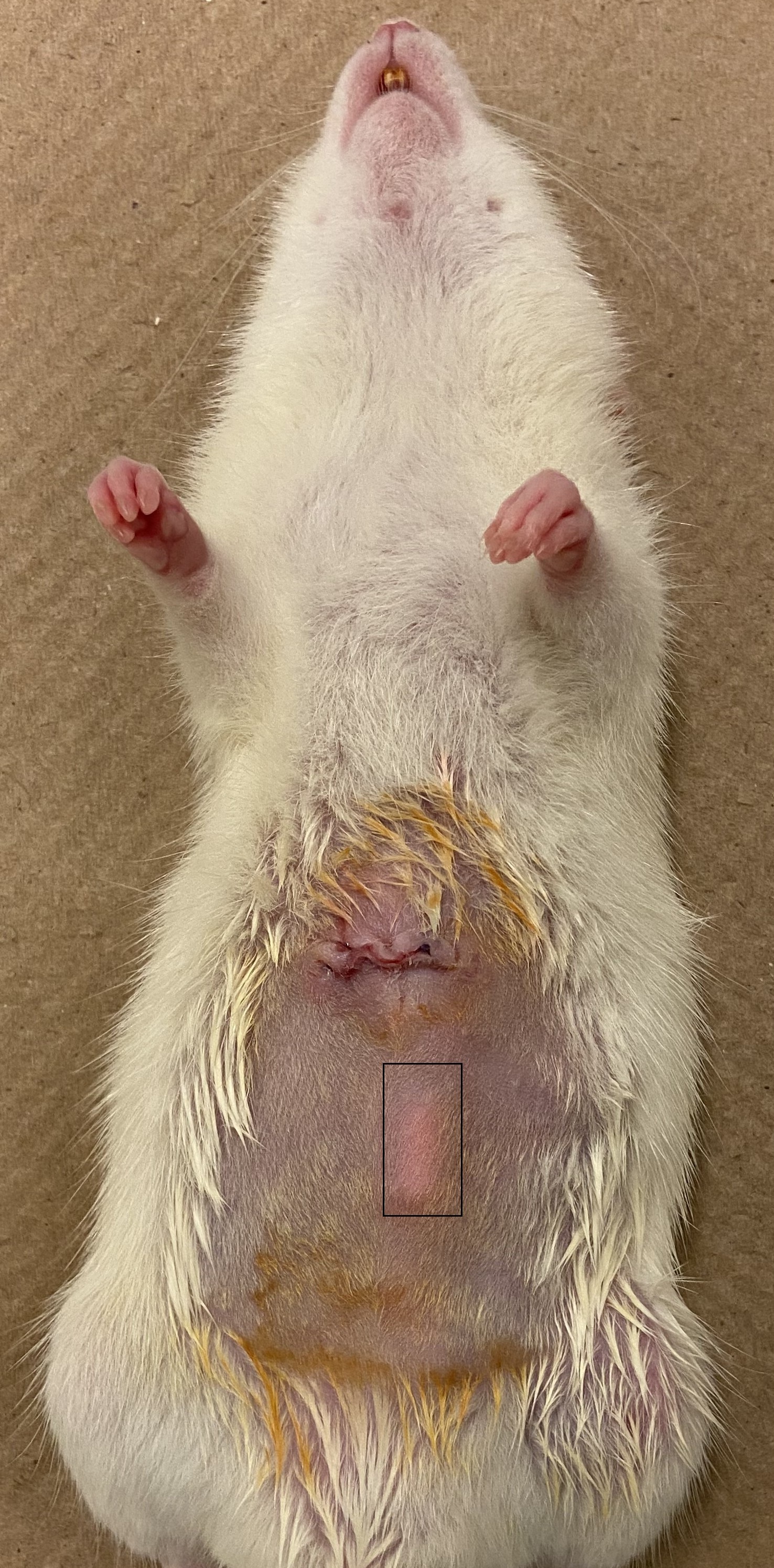

Supplement: Supplementary Figure 1 — Subcutaneous implantation of the Cell Pouch™ in the abdominal region of a rat. The image shows a representative rat after the Single-Channel Mini-Cell Pouch™ device placed in the subcutaneous abdominal space. The rectangular outline indicates the position of the implanted device beneath the skin. [file Supplementaryfile1.jpeg]
