# Supplementary material for: Enhancing post-operative hypothyroidism treatment: rat thyroid autotransplantation into a pre-vascularized, retrievable cell pouch™ device
Source: Front Endocrinol (Lausanne). 2025 Sep 17;16:1642916. doi: 10.3389/fendo.2025.1642916 (PMC12483921; doi:10.3389/fendo.2025.1642916)
Supplement: Supplementary Figure 2 — Comparative analysis of hematological and serum biochemical parameters. Blood tests were done at the baseline (one week before thyroidectomy and transplantation) and before the animals were euthanized (20 weeks post-thyroidectomy and transplantation). The non-parametric Wilcoxon Signed-Rank test was used to compare the values between the two time points. P values are indicated for each graph. Dash lines show the lower and upper normal limits. [file Supplementaryfile2.pptx]

## Slide 1
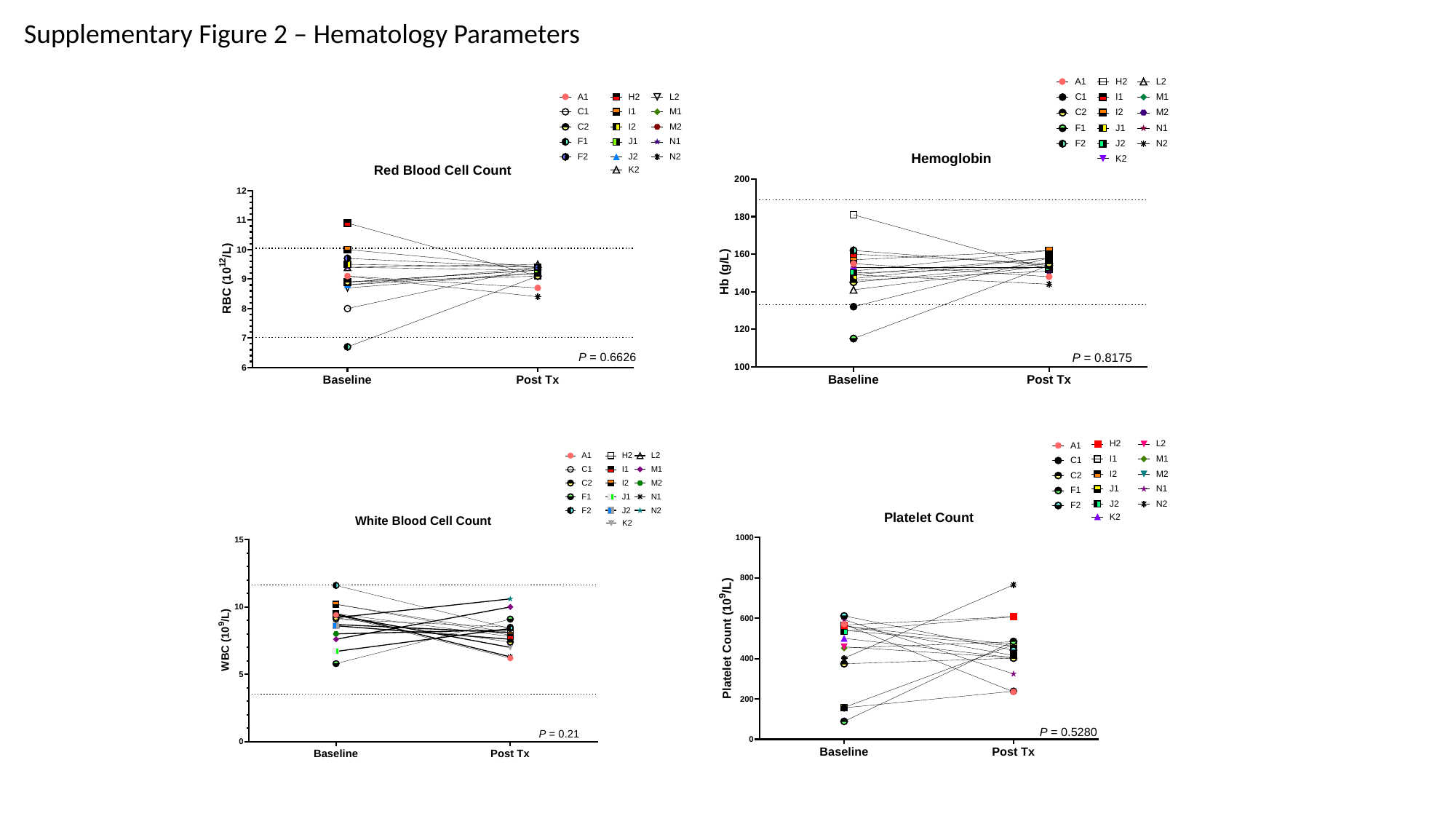

Supplementary Figure 2 – Hematology Parameters

## Slide 2
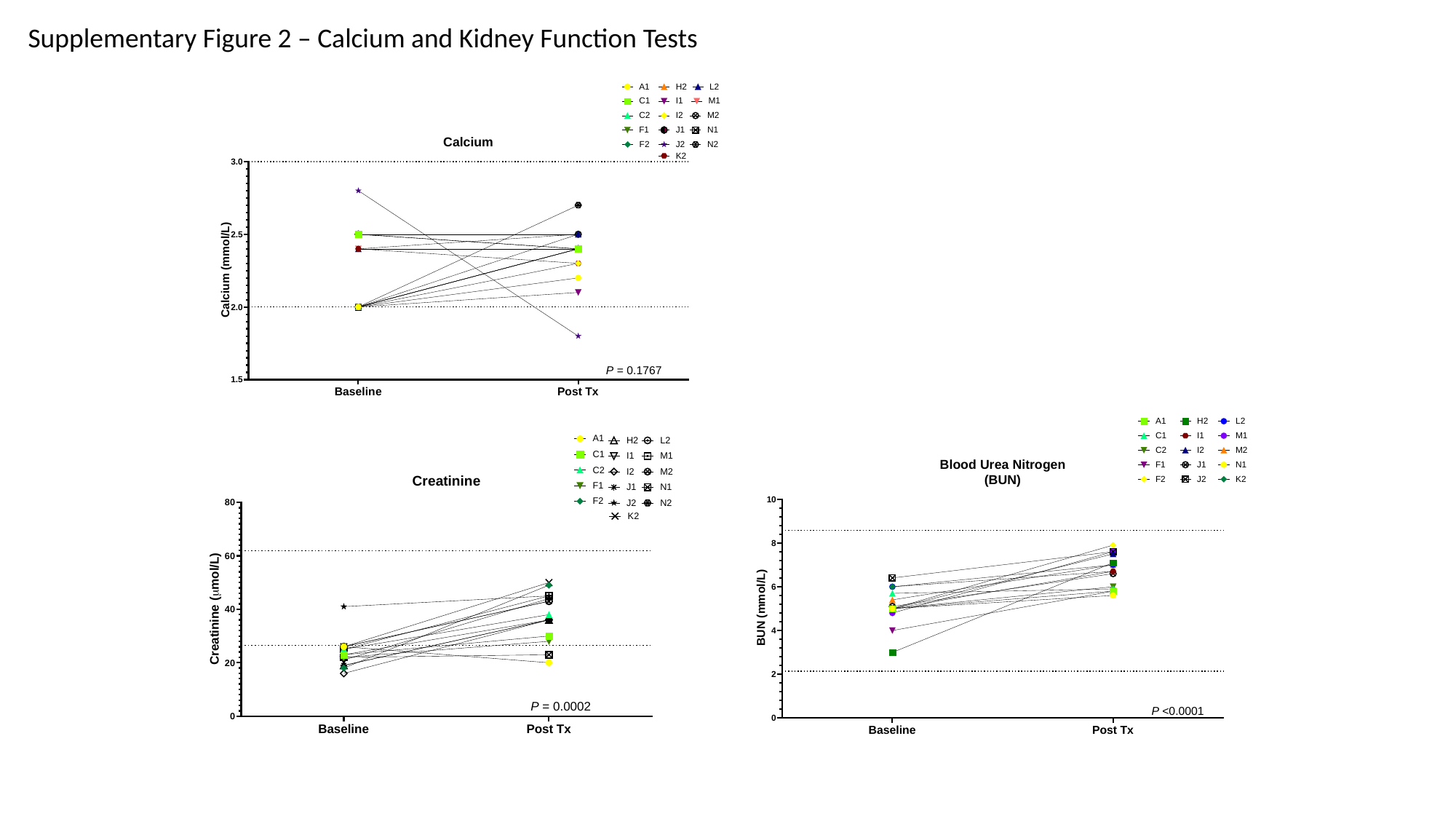

Supplementary Figure 2 – Calcium and Kidney Function Tests

## Slide 3
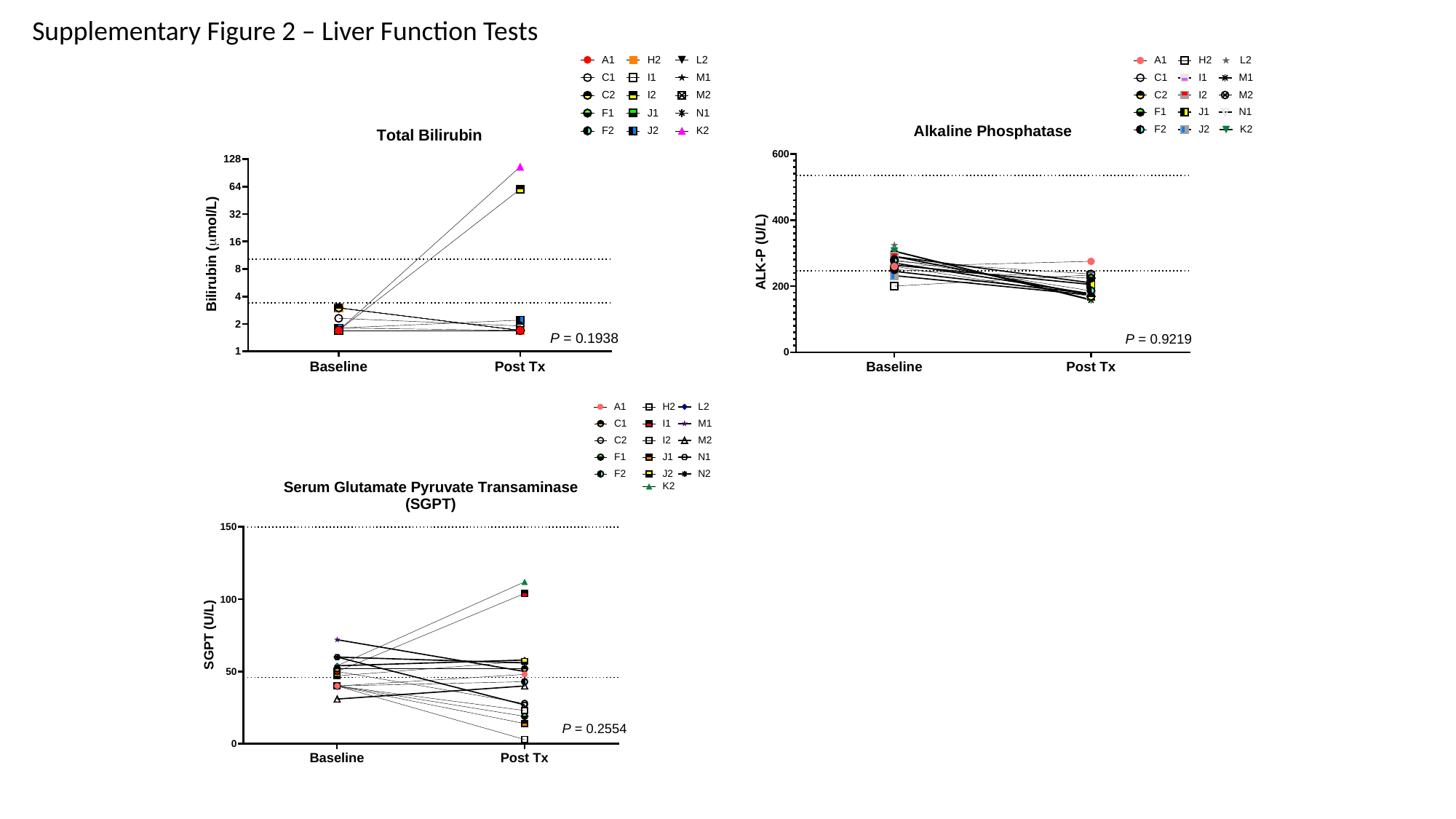

Supplementary Figure 2 – Liver Function Tests
